# AGING IN PLACE FOR OLDER HOMELESS VETERANS LIVING IN VA SUPPORTIVE HOUSING

**DOI:** 10.1093/geroni/igad104.3120

**Published:** 2023-12-21

**Authors:** Medha Maitra, Rebecca Brown

**Affiliations:** University of Georgia, Athens, Georgia, United States; University of Pennsylvania, Philadelphia, Pennsylvania, United States

## Abstract

Aging in place, defined as the ability to live comfortably, safely, and independently in one’s own home and community, is a priority for many older adults and a growing focus of research and policy efforts. However, little is known about perspectives on and experiences of aging in place among the growing population of older adults with experiences of homelessness, whose perspectives may differ from adults in the general population. Using qualitative interviews with veterans living in U.S. Department of Housing and Urban Development - VA Supportive Housing (HUD-VASH) apartments and focus groups with HUD-VASH staff members, we explored perspectives on and experiences of aging in place, including barriers and facilitators to aging in place in this setting.

